# Ocean acidification affects acid–base physiology and behaviour in a model invertebrate, the California sea hare (*Aplysia californica*)

**DOI:** 10.1098/rsos.191041

**Published:** 2019-10-09

**Authors:** Rebecca L. Zlatkin, Rachael M. Heuer

**Affiliations:** University of Miami Rosenstiel School of Marine and Atmospheric Science, Department of Marine Biology and Ecology, 4600 Rickenbacker Causeway, Miami, FL 33149, USA

**Keywords:** CO_2_, mollusc, carbon dioxide, climate change

## Abstract

Behavioural impairment following exposure to ocean acidification-relevant CO_2_ levels has been noted in a broad array of taxa. The underlying cause of these disruptions is thought to stem from alterations of ion gradients $\left(\text{HC}{\text{O}}_{3}^{-}/C{\text{l}}^{-}\right)$ across neuronal cell membranes that occur as a consequence of maintaining pH homeostasis via the accumulation of $\text{HC}{\text{O}}_{3}^{-}$. While behavioural impacts are widely documented, few studies have measured acid–base parameters in species showing behavioural disruptions. In addition, current studies examining mechanisms lack resolution in targeting specific neural pathways corresponding to a given behaviour. With these considerations in mind, acid–base parameters and behaviour were measured in a model organism used for decades as a research model to study learning, the California sea hare (*Aplysia californica*). Aplysia exposed to elevated CO_2_ increased haemolymph $\text{HC}{\text{O}}_{3}^{-}$, achieving full and partial pH compensation at 1200 and 3000 µatm CO_2_, respectively. Increased CO_2_ did not affect self-righting behaviour. In contrast, both levels of elevated CO_2_ reduced the time of the tail-withdrawal reflex, suggesting a reduction in antipredator response. Overall, these results confirm that Aplysia are promising models to examine mechanisms underlying CO_2_-induced behavioural disruptions since they regulate $\text{HC}{\text{O}}_{3}^{-}$ and have behaviours linked to neural networks amenable to electrophysiological testing.

## 1. Background

Ocean acidification is occurring at rates not observed in the last 300 million years. Average global oceanic CO_2_ levels are projected to increase from current levels of approximately 400 to approximately 940 µatm CO_2_ by the end of the century and approximately 1900 µatm CO_2_ by the year 2300 unless the rate of CO_2_ emissions is substantially curtailed [1–3]. This rapid rate of change has made predicting the sensitivity of organisms to future predicted CO_2_ levels a major focus of climate change research. Early studies focused heavily on calcifying invertebrates, reporting widespread impacts to calcification and growth [4]. Fish exposed to CO_2_ have exhibited alterations to mitochondrial pathways, intestinal base secretion and otolith growth [5–7].

In addition, impaired behaviour following CO_2_ exposure has been reported in more than approximately 130 studies to date in marine organisms at ocean acidification-relevant CO_2_ levels (less than 1900 µatm CO_2_). The majority of these studies have focused on marine fish, noting impairments to various endpoints including vision, olfaction, lateralization and learning [8–11], reviewed in [12]. Examination of behavioural disturbances has also been extended to invertebrates, where negative effects on predator defence behaviours have been observed [13–16].

The underlying cause of these behavioural disruptions is thought to result from the compensatory mechanism that allows fish and some active invertebrates to maintain pH homeostasis when exposed to elevated CO_2_. Following the onset of CO_2_ exposure, animals that are acid–base ‘regulators’ counter an initial drop in blood pH through the retention and/or uptake of $\text{HC}{\text{O}}_{3}^{-}$. This process allows acid–base regulators to correct pH to pre-exposure levels; however, both $\text{HC}{\text{O}}_{3}^{-}$ and PCO_2_ remain elevated [12,17,18]. This compensation mechanism is generally related to how ‘active’ an organism is, as higher metabolic rates (O_2_ consumption) necessitate higher rates of CO_2_ excretion [19]. The accumulation of $\text{HC}{\text{O}}_{3}^{-}$ in extracellular fluids is usually coupled with an equimolar decrease in Cl^−^ [18,20]. The resulting changes in extracellular and intracellular $\text{HC}{\text{O}}_{3}^{-}$ and Cl^−^ are thought to alter behaviour by attenuating the movement of these ions through the primary receptor (GABA_A_) responsible for background inhibitory responses in the vertebrate and invertebrate nervous system [11,21,22]. Thus, strong acid–base regulators with the ability to accumulate $\text{HC}{\text{O}}_{3}^{-}$ are hypothesized to be most at risk for behavioural disturbances [11].

Nilsson and colleagues [11] first implicated GABA_A_ receptor involvement in behavioural disruptions by treating CO_2_-impaired animals with gabazine, a GABA_A_ receptor antagonist. This treatment was found to reverse CO_2_-induced behavioural changes. Similar subsequent studies have continued to provide evidence for the involvement of GABA_A_ receptors using gabazine or muscimol (GABA_A_ receptor agonist) in fish [8,10,23–28] and in some invertebrates [13,29]. While this methodology has been seminal in providing a parsimonious explanation for altered behaviour and GABA_A_-receptor involvement in CO_2_-induced disruptions, future studies would benefit from two important considerations. First, although this proposed mechanism hinges on changes that occur following CO_2_ compensation, few studies have measured acid–base parameters in a marine species while also measuring behaviour [30–35]. Such measurements would be especially important in invertebrates, where there is more inherent variation in acid–base regulatory ability [19,36,37]. For example, sea urchins (*Paracentrotus lividus*) retain $\text{HC}{\text{O}}_{3}^{-}$ to defend pH, while mussels (*Mytilus edulis*) do not, and experience an acidosis when exposed to the same CO_2_ level (1480 µatm CO_2_) [38]. Second, although crucial in implicating the GABA_A_ receptor, immersing an animal in seawater containing a GABA_A_ receptor pharmacological agent lacks resolution in targeting specific behaviours and could induce effects on unintended targets [23]. In addition, there has been little exploration of potential alternative or additional mechanisms in CO_2_-induced behavioural disruptions [39,40]. Finally, although the majority of CO_2_ behavioural studies are performed on fish, the vertebrate nervous system is complex, making it difficult to link a particular behaviour to specific neural networks.

To address these limitations, we propose that future research examining the behavioural impacts of CO_2_ would benefit from identifying a model organism well-suited for both acid–base balance and neurophysiological studies. The ideal study organism would meet three criteria: (1) a simple and well-mapped nervous system, (2) reproducible behavioural assays, and (3) an acid–base ‘regulator’ profile, with the ability to accumulate $\text{HC}{\text{O}}_{3}^{-}$ to defend pH. The California sea hare (*Aplysia californica)*, referred to herein as ‘Aplysia’, is widely known to meet the first two criteria perfectly and has been used for decades as a biomedical research model to study the cellular basis of learning [41].

Since the ability to acid–base regulate has been linked to behavioural disruptions, measuring the baseline CO_2_ acid–base response in Aplysia is a necessary step in assessing their feasibility as a model for CO_2_ behavioural research. The first objective of the present study was to examine acid–base parameters in haemolymph from Aplysia exposed to either control (approx. 400), 1200 or 3000 µatm CO_2_. Since Aplysia are not sessile invertebrates, it was hypothesized that they would exhibit an acid–base ‘regulator’ profile, and actively retain $\text{HC}{\text{O}}_{3}^{-}$ to defend pH following CO_2_ exposure. The second objective of this study was to examine the impacts of elevated CO_2_ on two simple behaviours with well-characterized neural networks [42], the righting reflex and the tail-withdrawal reflex. Righting is important for orientation and reattachment to substrate, while the tail-withdrawal reflex is an antipredator response that activates muscles used in escape responses [42–44]. Elevated CO_2_ was expected to alter behaviour, as noted in previous studies. Notably, the chosen behavioural assays and CO_2_ levels are environmentally relevant for Aplysia living in the intertidal zone of the North American Pacific coast [45]. Ultimately, this study marks the first step in assessing Aplysia as a potential model for future studies of CO_2_-induced behavioural disruptions in marine organisms, including exploration of the GABA_A_ hypothesis in addition to potential alternative mechanisms.

## 2. Material and methods

### 2.1. Animal care and experimental exposure

Aplysia (*Aplysia californica)*, hatchery-reared from egg masses of wild-caught animals, were provided by the National Resource for Aplysia (National Institute of Health Grant P40OD010952) at the University of Miami Rosenstiel School of Marine and Atmospheric Science. Prior to use in experiments, Aplysia were fed ad libitum with red macroalga *Gracilaria ferox* and *Agardhiella subulata* [46] and were kept in 16 l tanks with a seawater flow rate of approximately 1.3 l min^−1^ at approximately 15°C.

During experimentation, Aplysia were acclimated to either control (400), 1200 or 3000 µatm CO_2_ for acid–base (*n* = 2–3 tank replicates, 3–5 animals/tank) and behavioural experiments (*n* = 4–7 tanks, 2–5 animals/tank). These acclimations were performed in 16 l tanks with flow-through seawater (0.6 l min^−1^, 15°C). Animals were exposed for either 4 or 11 days to each CO_2_ level. Since day of exposure (4 versus 11) did not significantly impact any measured endpoint (see below), exposure duration is referred to as 4–11 days throughout the manuscript. These time periods have previously been sufficient to reach a stable $\text{HC}{\text{O}}_{3}^{-}$ accumulation for CO_2_ compensation [47]. In addition, 4 days is close to the 5-day exposure period previously demonstrated to induce behavioural disruptions in other invertebrates [13,14]. Animals were permitted to feed on the first day of the exposure but food was subsequently withheld approximately 96 h prior to experimental testing. Animals that experienced 11-day exposures were subjected to the same approximately 96 h fasting period. Animals remained immersed in seawater throughout the experimental period. Animals used in experiments were approximately 10–11 months of age and weighed 90–110 g (electronic supplementary material, table S1).

### 2.2. Seawater CO_2_ manipulation

Desired PCO_2_ levels were achieved using a CO_2_ negative feedback system as previously described (Loligo Systems, Denmark) [6]. First, a standard curve was made by determining the relationship between known gas standards and seawater pH. Using this relationship, a pH set-point corresponding to each desired PCO_2_ level was calculated, and 100% CO_2_ was slowly bubbled into flow-through, aerated tanks to achieve the chosen PCO_2_ level. The pH electrode and meter (WTW Sentix H electrode and 3310 meter) corresponding to each experimental tank were connected to CapCTRL software that delivered CO_2_ using solenoid valves controlled by a DAQ-M digital relay instrument (Loligo Systems). Validation of desired PCO_2_ values was achieved using pH_NBS_ and total CO_2_ (TCO_2_) and was performed approximately two times per experiment. Measurements of pH_NBS_ were recorded multiple times per week using an independent pH electrode and meter (Radiometer PHC3005 electrode, ThermoFisher Orion Star A221 meter). A Corning 965 CO_2_ analyser (Corning Diagnostics) was used to measure TCO_2_. To calculate PCO_2_ and titratable alkalinity (TA), values of pH_NBS_ and TCO_2_, were entered into CO2SYS [48]. Calculated PCO_2_ values for control, 1200 and 3000 μatm CO_2_ are reported in electronic supplementary material, table S2. Temperature and salinity were measured approximately three times per week (WTW 3310 meter and TetraCon 325; electronic supplementary material, table S2).

### 2.3. Objective 1: Haemolymph acid–base balance and ion measurements

Extracellular haemolymph was sampled by inserting a 500 µl gas-tight glass syringe (Hamilton) towards the posterior and alongside the foot of the animal and gently withdrawing fluid. Haemolymph was measured immediately for extracellular pH (pH_e_) using a custom glass chamber fitted around a needle pH microsensor attached to pH-1 Micro meter (Loligo Systems). The pH microsensors were pre-calibrated from the manufacturer and were corrected after verification with a known pH_NBS_ value from sterile seawater. This sterile seawater was used to flush out the pH chamber in between sample measurements and was measured using Radiometer PHC3005 pH electrode attached to a ThermoFisher Orion Star A221 meter. Haemolymph from the same animal was measured for TCO_2_ (Corning 965, Corning Diagnostics). $\text{HC}{\text{O}}_{3}^{-}$ and PCO_2_ were calculated from TCO_2_ and pH using the Henderson–Hasselbach equation using an established solubility constant (αCO_2_) and dissociation constant (pK) for carbonic acid [49].

### 2.4. Objective 2: General behavioural assay protocols

For all behavioural assays, animals were gently removed from their experimental tank and placed carefully in the bottom of test tanks in water at their respective acclimation PCO_2_ level. Tanks were 16 l and had a depth of 16 cm. In all assays, animals were given a 5 min acclimation time to become accustomed to the test tank prior to commencing behavioural tests. All assays were recorded on video and the experimenter was blind to the experimental treatment both during experiments and video analyses. In some cases, animals were tested in one of the two behaviour assays on the 4th day of exposure, returned to acclimation tanks, then tested on the 11th day for the second behaviour. The order of behaviours tested was altered. Based on previous studies, even repeated stimuli or noxious stimuli do not elicit long-term memory formation (animals retested on day 7) [50–53]. Accordingly, there was no reason to suspect that the mild stimulus in the present study would impact animals receiving a second behavioural test. In both assays, animals remaining in a contracted state for more than 1 min or animals that inked during tests were eliminated from analyses as ‘non-participators’. In previous studies, extreme stress has been shown to lead to tachycardia and suppressed reflex activity [54], and inking is considered a ‘high-threshold, all or none’ behaviour [55]. This criteria resulted in removal of five control, seven 1200 µatm and eight 3000 µatm animals from the tail-withdrawal assay. One animal was removed from the 1200 µatm and the 3000 µatm treatments during the righting assay.

### 2.5. Righting behavioural assay

Protocols followed those outlined in a previous study [42]. Following the 5 min acclimation period, the animal was gently lifted to the top of the water column and released while on its side. The start time of the reflex occurred the moment the animal made contact with the bottom of the tank. The time from bottom contact to when the animal returned to an upright position and initiated its first crawling was recorded as righting time. The assay was performed in triplicate with a rest period of 5 min between trials [42,43]. The data was summarized for each individual as the mean of the triplicate measurements for the reflex time.

### 2.6. Tail-withdrawal behavioural assay

Protocols followed those outlined in previous studies [42,56]. Following the acclimation period, the animal was carefully lifted off the test tank bottom, and gently held by the experimenter as close to the tank bottom as possible without allowing the animal to adhere to the bottom (approx. 1 cm). At this point, the length from the tip of the tail to the top of the head was measured and recorded as the resting length, using a transparent ruler lying next to the animal in the bottom of the tank. The animal was then placed on the bottom of the tank and a blunted 20G needle was pressed onto the tip of the animal's tail (approx. 50–70° angle) for one second to depress the tissue against the test tank bottom to a depth approximately half the thickness of the tail. This depression caused the tail to withdraw and represented the starting time of the reflex. At maximal contraction, the total length of the animal from the tip of the tail to the top of the head was noted using the ruler. Relaxation of the tail to approximately 50% of the original tail length signified the end of the reflex. The reflex was measured in triplicate with rest intervals of 10 min between each trial [42].

### 2.7. Statistical analysis

Linear mixed effect (LME) models were used to test for responses to CO_2_ exposure levels for the time to complete the righting reflex and the time to complete the tail-withdrawal reflex. These models included CO_2_ level and day of exposure as fixed factors, and tank as a random factor. Tukey's *post hoc* tests with a Holm-adjusted *p*-value was used to compare means between CO_2_ exposure levels. The righting time and the tail-withdrawal reflex time data were log-transformed prior to analysis. A general linear model was used in instances where inclusion of tank as a random factor in mixed models resulted in overfit, using treatment and day as fixed factors. This applied to the per cent of the tail withdrawn in the tail-withdrawal reflex, haemolymph pH_e_, haemolymph $\text{HC}{\text{O}}_{3}^{-}$ and haemolymph PCO_2_. The per cent of the tail withdrawn in the tail-withdrawal reflex was arcsin transformed prior to statistical analysis. All models were conducted in R v. 3.5.2 [57] using the lme4 and lmerTest packages [58,59], and *post hoc* testing was conducted using the multcomp package [60]. Significance was determined at *p* < 0.05 for all tests and all values are presented as means ± s.e.m. Figures were made using SigmaPlot 13.0 and presented as treatment means pooled across days of exposure, since day of exposure was not significant in any test.

## 3. Results

### 3.1. Physiological measurements

**Figure 1.**
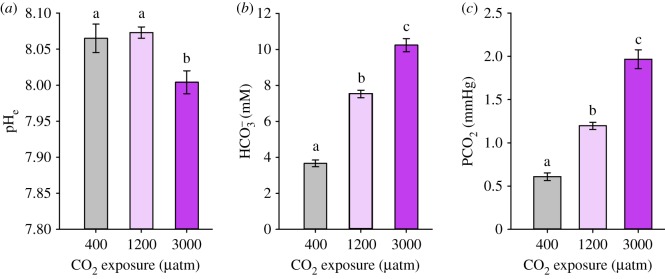
Haemolymph (*a*) pH_e_, (*b*) $\text{HC}{\text{O}}_{3}^{-}$ and (*c*) PCO_2_ in Aplysia (*Aplysia californica*) exposed to either control (400), 1200 µatm CO_2_ or 3000 µatm CO_2_ for 4–11 days. Values are reported as means ± s.e.m; *n* = 10–11. Means that share the same letter are not significantly different (*p* < 0.05).

For all parameters, the day of testing was not significant, so results are presented across CO_2_ treatments. Haemolymph pH_e_ was significantly affected by CO_2_ exposure (figure 1*a*; *F*_2,29_ = 5.94, *p* = 0.007), but was not affected by the day of testing (*F*_1,29_ = 0.248, *p* = 0.622). Accordingly, *post hoc* comparisons on the effect of CO_2_ exposure on haemolymph pH_e_ reflected pooled values across days of exposure. Aplysia exposed to CO_2_ for 4–11 days showed a significant reduction in haemolymph pH_e_ at 3000 (*t* = −2.736, *p* = 0.021), but not at 1200 µatm CO_2_ when compared with controls (figure 1*a*; *t* = 0.373, *p* = 0.712). As expected, Aplysia showed evidence of a compensatory response via the accumulation of $\text{HC}{\text{O}}_{3}^{-}$ (figure 1*b*). $\text{HC}{\text{O}}_{3}^{-}$ was significantly affected by CO_2_ exposure (*F*_2,27_ = 157.53, *p* < 0.001), but was not affected by day of testing (*F*_1,27_ = 2.15, *p* = 0.15). *Post hoc* comparisons revealed significant differences in $\text{HC}{\text{O}}_{3}^{-}$ between all three CO_2_ levels (figure 1*b*, all *p* < 0.001). pCO_2_ increased significantly with CO_2_ exposure (*F*_2,27_ = 87.41, *p* < 0.001), but was also not affected by day of testing (*F*_1,27_ = 0.036, *p* = 0.850). *Post hoc* testing revealed significant differences in pCO_2_ between all three CO_2_ levels (figure 1*c*, all *p* < 0.001).

The relationship between haemolymph $\text{HC}{\text{O}}_{3}^{-}$ and PCO_2_ exposure was not perfectly linear, which probably accounts for incomplete pH compensation at 3000 µatm CO_2_ (electronic supplementary material, figure S1).

### 3.2. Behavioural responses

**Figure 2.**
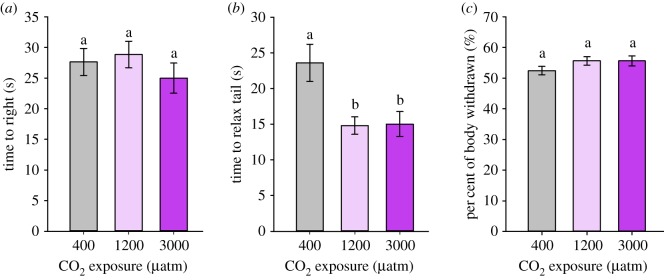
Behavioural analysis in Aplysia (*Aplysia californica*) exposed to either control (400), 1200 µatm CO_2_ or 3000 µatm CO_2_ for 4–11 days. (*a*) Righting reflex (*n* = 13–16), (*b*) tail-withdrawal reflex (TWR) amount of time to relax the tail to 50% of original length and (*c*) TWR percentage of starting body length withdrawn following tail touch (*n* = 19, 17, 16 for control, 1200 µatm CO_2_ and 3000 µatm CO_2_, respectively). All values are reported as means ± s.e.m. Means that share the same letter are not significantly different (*p* < 0.05).

Aplysia exposed to CO_2_ displayed no difference in the time to right when compared with control animals (figure 2*a*; *F*_1,13_ = 0.411, *p* = 0.533). The day of testing did not affect the righting response (*F*_1,12_ = 0.002, *p* = 0.964). Tail-withdrawal time was significantly affected by increased CO_2_ exposure (*F*_2,15_ = 4.52, *p* = 0.029), but was not affected by the day of testing (*F*_1,16_ = 0.04, *p* = 0.84). Accordingly, *post hoc* comparisons on the effect of CO_2_ exposure on tail-withdrawal reflex time reflected pooled values across days of exposure. Animals exposed to 1200 and 3000 µatm CO_2_ relaxed their tail approximately 36–37% faster than control animals (figure 2*b*; *z* = −2.521, *p* = 0.027, *z* = −2.612, *p* = 0.027, respectively). High CO_2_-exposed groups did not show a significant difference from one another (*z* = 0.134, *p* = 0.893). The percentage of body length withdrawn following tail depression exhibited no significant differences with treatment or day (figure 2*c*; treatment: *F*_1,49_ = 2.58, *p* = 0.12, day: *F*_1,49_ = 1.36, *p* = 0.25).

## 4. Discussion and conclusion

Aplysia exposed to elevated CO_2_ (1200 and 3000 µatm CO_2_) were able to accumulate significantly higher levels of $\text{HC}{\text{O}}_{3}^{-}$ in haemolymph following a 4–11 day exposure period (figure 1*b*). This compensatory effort led to complete pH defence at 1200 µatm CO_2_, an ocean acidification-relevant level close what is predicted globally by year 2100 (940 µatm CO_2_ under business as usual [2]) (figure 1*a*; 1200 µatm CO_2_). Of the two behavioural responses tested, tail withdrawal was impacted by high CO_2_ exposure, as hypothesized, whereas righting was not (figure 2).

It has long been known that invertebrates show more inherent variation in acid–base regulatory ability than fish. Generally, active invertebrates tend to show a stronger $\text{HC}{\text{O}}_{3}^{-}$ buffering capacity, while less active invertebrates may experience metabolic suppression associated with a decline in pH [19,37]. In studies addressing acid–base status of invertebrates at ocean acidification-relevant CO_2_ levels (less than approximately 2000 µatm CO_2_), sea urchins (*Paracentrotus lividus, Echinometra mathaei*, *Tripneustes ventricosus*) [36,38,61–63], Arctic spider crabs (*Hyas araneus*) [35], velvet swimming crabs (*Necora puber*) [64] and shore crabs (*Carcinus maenas*) [33] all accumulate $\text{HC}{\text{O}}_{3}^{-}$ to correct an acidosis. In contrast, blue mussels (*Mytius edulis*) [38], king scallops (*Pecten maximus*) [34], northern sea urchins (*Strongylocentrotus drobachiensis*) [65], sea stars (*Asteria rubens, Leptasterias polaris*) [33,66], slate pencil sea urchins (*Eucidaris tribuloides*) [36] and Arctic spider crabs at higher CO_2_ levels (3000 µatm CO_2_) [35] show incomplete or an absence of $\text{HC}{\text{O}}_{3}^{-}$ accumulation that is often insufficient in maintaining pH during high CO_2_ exposure. The diversity in acid–base responses to CO_2_ seen among invertebrates offers a fruitful avenue for studies of the mechanistic underpinnings of disturbed behaviour. Responses in animals showing regulatory and non-regulatory responses can be studied in the same species using Aplysia. It is clear that they regulate pH at lower CO_2_ levels (1200 µatm CO_2_) but cannot maintain this response at higher CO_2_ levels (3000 µatm CO_2_) (figure 1*a*; electronic supplementary material, figure S1).

Similar to acid–base regulatory ability, the behavioural responses of invertebrates have been variable. In the present study, CO_2_ exposure did not alter the self-righting response of Aplysia (figure 2*a*). This mirrors self-righting results of CO_2_-exposed gastropod molluscs (*Gibberulus gibbosus*) [13] and sea stars (*Asteria rubens*) [33]. Some studies have noted a faster righting time with elevated CO_2_, in brittlestars (*Ophiura ophiura*) at higher CO_2_ levels (corresponding to pH 7.3) [67], and in the Chilean abalone (*Concholepas concholepas*) [68]. In one case, righting time has been shown to increase with elevated CO_2_ exposure in a marine gastropod (*Margarella antarctica*) [69], and there was a trend of an increase in the cone snail (*Conus marmoreus*; *p* = 0.052) [70]. The tail withdrawal, a defence mechanism elicited by Aplysia, showed a significant decrease in reflex time at elevated CO_2_ levels (figure 2*b*), taking more time to relax the tail to half its original length after maximum contraction, but showed no change to the magnitude of the response (% body length contracted; figure 2*c*). Animals exposed to elevated CO_2_ relaxed their tail approximately 37% faster compared with control animals. The decrease in the timing of the tail-withdrawal reflex could suggest a decline in antipredator response or increased boldness, findings that have been observed across taxa [9,13,14,16]. For example, the marine snail *G. gibbosus* jumped away from a predator cue less frequently and with increased latency when exposed to elevated CO_2_ [13]. Similarly, flight behaviour of the black turban snail (*T. funebralis*) was reduced with increasing CO_2_, albeit at higher CO_2_ levels corresponding to a pH of 7.1 [15].

Given the ubiquity of CO_2_-induced behavioural disruptions across taxa, a common neural mechanism has been proposed, where altered $\text{HC}{\text{O}}_{3}^{-}$ and Cl^−^ ion gradients resulting from efforts to maintain pH homeostasis are presumed to change the function of the GABA_A_ receptor [11]. Despite the proposed link between acid–base regulatory ability and behavioural disruptions in marine organisms, this study represents one of few that have measured both parameters in the same species at ocean acidification-relevant CO_2_ levels [30–35]. Although Aplysia experienced an acidosis at 3000 µatm CO_2_, they were still able to accumulate $\text{HC}{\text{O}}_{3}^{-}$ at both CO_2_ levels. Based on extracellular measurements, this change could alter neuronal gradients and possibly explained shortened time to tail relaxation. However, in other invertebrate studies, sea stars unable to elevate $\text{HC}{\text{O}}_{3}^{-}$ [32,33] and crabs able to elevate $\text{HC}{\text{O}}_{3}^{-}$ both showed no difference in righting [35]. Scallops showing an acidosis with very limited $\text{HC}{\text{O}}_{3}^{-}$ accumulation showed significant impacts on clapping performance [34]. The lack of consistency across studies and in Aplysia in the current study may seem difficult to reconcile. The source of variation could stem from a number of factors including differential intracellular pH regulation or behavioural compensatory mechanisms. In addition, these variations may reflect that certain behaviours are not GABA-mediated. It is clear that the field would benefit from more measurements of acid–base parameters in species showing behavioural disruptions to help resolve these discrepancies.

Although the involvement of the GABA_A_ receptor was not directly tested in the present study, GABA_A_ receptor involvement in CO_2_-induced behavioural disruptions have been demonstrated in some fish [8,10,23–28] and invertebrates [13,29]. These studies have largely implicated GABA_A_ receptor involvement using whole-animal exposure to pharmacological agents targeting GABA_A_. This method has been fundamental in establishing the proposed mechanism, but lacks resolution in targeting specific mechanisms responsible for a given behavioural disturbance. Since Aplysia accumulate $\text{HC}{\text{O}}_{3}^{-}$ and show a significant behavioural disruption at both tested CO_2_ levels, they are an ideal candidate for obtaining a better understanding of mechanisms underlying CO_2_ behavioural impairment. Findings from the present study combined with decades of research examining the electrophysiological basis of learning means that methods to link well-characterized neural networks to specific behaviours are already established. For many reflexes, including the CO_2_-impacted tail-withdrawal reflex, the reflex can be elicited in *in vitro* preparations where the specific neural network for a given reflex is isolated from the animal [56]. In addition, Aplysia neurons are large and amenable to patch clamp techniques, where individual cells and/or specific transporters can be investigated. While the specific role of the GABA_A_ is not well-studied in the context of the CO_2_-impacted tail-withdrawal reflex, gamma-aminobutyric acid (GABA) has been localized to certain areas in the pedal ganglia [71], which innervates the tail [42,72]. Furthermore, Aplysia neurons from a number of regions including the pleural ganglia (also involved in the tail-withdrawal reflex), have shown both excitatory and inhibitory currents with the application of GABA and were found to be reactive to GABA_A_ receptor antagonists [73].

In summary, we believe all of the advantages of using Aplysia as a biomedical research model for learning could be applied to ocean acidification research. Aplysia meet three important criteria (1–3). In addition to simple and well-mapped nervous systems (1), there are established and reproducible behavioural assays (2) that can be applied to examine all major forms of learning including habituation, sensitization, classical conditioning and operant conditioning [74]. Most importantly, the present study demonstrates that Aplysia accumulate $\text{HC}{\text{O}}_{3}^{-}$ at an ocean acidification-relevant CO_2_ level (3). These three criteria allow for further exploration of the proposed link between acid–base regulatory ability and behaviour, including detailed testing of GABA_A_ hypothesis.

## Supplementary Material

Table S1: Body mass

Reviewer comments

## Supplementary Material

Table S2: Water chemistry

## Supplementary Material

Figure S1: Bicarbonate as a function of CO2 exposure

## Data accessibility

Our data are deposited at the Dryad Digital Repository: https://doi.org/10.5061/dryad.7pd654v [75].
