# Supplementary material for: Ocean acidification affects acid–base physiology and behaviour in a model invertebrate, the California sea hare (Aplysia californica)
Source: R Soc Open Sci. 2019 Oct 9;6(10):191041. doi: 10.1098/rsos.191041 (PMC6837219; doi:10.1098/rsos.191041)
Supplement: Table S1: Body mass [file rsos191041supp1.docx]

**Ocean acidification affects acid-base physiology and behaviour in a model invertebrate, the California sea hare (*Aplysia californica*)**

Rebecca L. Zlatkin^1^ and Rachael M. Heuer^1*^

^1^University of Miami Rosenstiel School of Marine and Atmospheric Science, 4600 Rickenbacker Causeway, Miami, FL 33149

*****corresponding author, rheuer@rsmas.miami.edu

**Supplementary Table 1:** Body mass in Aplysia (*Aplysia californica*) exposed to either control (400), 1200 μatm CO_2_ or 3000 μatm CO_2_ for 4-11 days. Means ± s.e.m are presented for acid-base and behavioural experimental objectives.

|  | CO_2_ level  (μatm CO_2_) | Animal mass (g) |
| --- | --- | --- |
| Acid-base parameters | Control | 110.2 ± 3.5 |
|  | 1200 | 100.4 ± 2.9 |
|  | 3000 | 100.1 ± 4.0 |
| Behavioural experiments-Tail withdrawal reflex | Control | 94.7 ± 3.9 |
|  | 1200 | 91.0 ± 4.8 |
|  | 3000 | 92.4 ± 3.7 |
| Behavioural experiments-Righting | Control | 92.1 ± 3.5 |
|  | 1200 | 99.6 ± 3.7 |
|  | 3000 | 90.8 ± 4.1 |
