# Supplementary material for: Ocean acidification affects acid–base physiology and behaviour in a model invertebrate, the California sea hare (Aplysia californica)
Source: R Soc Open Sci. 2019 Oct 9;6(10):191041. doi: 10.1098/rsos.191041 (PMC6837219; doi:10.1098/rsos.191041)
Supplement: Table S2: Water chemistry [file rsos191041supp2.docx]

**Ocean acidification affects acid-base physiology and behaviour in a model invertebrate, the California sea hare (*Aplysia californica*)**

Rebecca L. Zlatkin^1^ and Rachael M. Heuer^1*^

^1^University of Miami Rosenstiel School of Marine and Atmospheric Science, 4600 Rickenbacker Causeway, Miami, FL 33149

*****corresponding author, rheuer@rsmas.miami.edu

**Supplementary Table 2:** Water chemistry parameters for acid-base and behavioural experimental objectives in Aplysia (*Aplysia californica*) exposed for 4-11 days to either control (400), 1200 μatm CO_2_ or 3000 μatm CO_2_. Values are presented as means ± s.e.m. PCO_2_ was calculated using values of pH_NBS_, TCO_2_, salinity, and temperature in CO2SYS using the constants K1 from Merbach et al 1973 refit by Dickson and Miller (1987), and Dickson for KHSO_4_ [1].

|  | CO_2_ level  (µatm CO_2_) | Salinity | Temperature (°C) | pH_NBS_ | *p*CO_2_  (µatm CO_2_) | TA  (μmol/kg) | TCO_2_  (μmol/kg) |
| --- | --- | --- | --- | --- | --- | --- | --- |
| Acid-base | Control | 32.3 ± 0.5 | 15.0 ± 0.04 | 8.16 ± 0.02 | 426.1 ± 17.1 | 2293 ± 53.3 | 2096.0 ± 40.9 |
|  | 1200 | 32.2 ± 0.5 | 15.0 ± 0.05 | 7.76 ± 0.01 | 1234.4 ± 58.5 | 2393.2 ± 91.4 | 2342.9 ± 80.05 |
|  | 3000 | 32.6 ± 0.5 | 14.9 ± 0.09 | 7.41 ± 0.01 | 2878.1 ± 316.8 | 2411.2 ± 90.4 | 2474.4 ± 103.4 |
| Tail withdrawal | Control | 32.9 ± 0.4 | 14.9 ± 0.05 | 8.17 ± 0.008 | 411.3 ± 18.0 | 2284.5 ± 47.9 | 2081.8 ± 47.0 |
|  | 1200 | 33.1 ± 0.4 | 14.9 ± 0.08 | 7.77 ± 0.006 | 1146.4 ± 23.7 | 2394.8 ± 41.2 | 2334.7 ± 39.8 |
|  | 3000 | 33.1 ± 0.4 | 14.9 ± 0.07 | 7.36 ± 0.02 | 3014.6 ± 64.6 | 2435.7 ± 23.3 | 2504.8 ± 24.7 |
| Righting | Control | 33.3 ± 0.4 | 14.9 ± 0.1 | 8.18 ± .01 | 400.0 ± 24.9 | 2289.6 ± 56.2 | 2078.6 ± 58.1 |
|  | 1200 | 33.6 ± 0.5 | 14.8 ± 0.2 | 7.78 ± 0.007 | 1166.3 ± 37.7 | 2417.4 ± 54.1 | 2356.0 ± 52.3 |
|  | 3000 | 33.1 ± 0.1 | 14.9 ± 0.1 | 7.38 ± 0.005 | 2927.1 ± 40.7 | 2423.5 ± 22.1 | 2487.9 ± 23.3 |

[1] Pierrot, D., Lewis, E. & Wallace, D. 2006 MS Excel program developed for CO2 system calculations. *ORNL/CDIAC-105a. Carbon Dioxide Information Analysis Center, Oak Ridge National Laboratory, US Department of Energy, Oak Ridge, Tennessee*.
