# Supplementary material for: Ocean acidification affects acid–base physiology and behaviour in a model invertebrate, the California sea hare (Aplysia californica)
Source: R Soc Open Sci. 2019 Oct 9;6(10):191041. doi: 10.1098/rsos.191041 (PMC6837219; doi:10.1098/rsos.191041)
Supplement: Figure S1: Bicarbonate as a function of CO2 exposure [file rsos191041supp3.docx]

**Ocean acidification affects acid-base physiology and behaviour in a model invertebrate, the California sea hare (*Aplysia californica*)**

Rebecca L. Zlatkin^1^ and Rachael M. Heuer^1*^

^1^University of Miami Rosenstiel School of Marine and Atmospheric Science, 4600 Rickenbacker Causeway, Miami, FL 33149

*****corresponding author, rheuer@rsmas.miami.edu


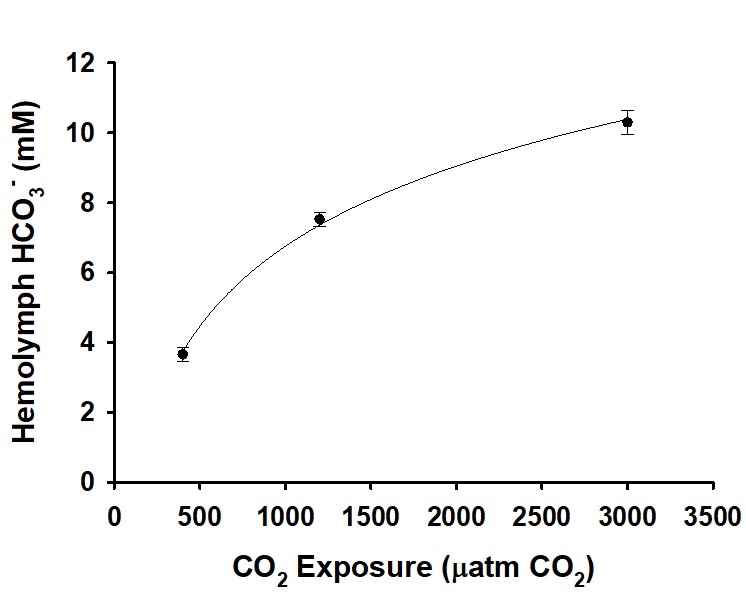


**Supplementary Figure S1**: Hemolymph HCO_3_^-^ as a function of CO_2_ exposure following 4-11 days of exposure. y = 3.30ln(x) - 16.06, R² = 0.998. Error bars represent SEM.
